# Characterization of Lignocellulosic Byproducts from the Portuguese Forest: Valorization and Sustainable Use

**DOI:** 10.3390/ma18204716

**Published:** 2025-10-14

**Authors:** Morgana Macena, Luísa Cruz-Lopes, Lucas Grosche, Isabel Santos-Vieira, Bruno Esteves, Helena Pereira

**Affiliations:** 1 CERNAS-IPV Research Centre, Polytechnic University of Viseu, Campus Politécnico, Repeses, 3504-510 Viseu, Portugal; bruno@estgv.ipv.pt; 2 CEF—Forest Research Centre, TERRA Associate Laboratory, School of Agriculture, University of Lisbon, 1349-017 Lisboa, Portugal; hpereira@isa.utl.pt; 3 4iTec Lusitânia S.A., Lugar do Pombal, Zona Industrial do Salgueiro, 3530-259 Mangualde, Portugal; lucas.grosche@4iteclusitania.pt; 4 CICECO—Aveiro Institute of Materials, Department of Chemistry, University of Aveiro, 3810-193 Aveiro, Portugal; ivieira@ua.pt

**Keywords:** pines, acacia, biomass, physical-chemical characterization, bioadsorbents, forestry residues

## Abstract

The increasing emphasis on environmental sustainability has placed biomass as a versatile and renewable resource, while the management and disposal of forest byproducts remain a significant challenge. This study explores the valorization of forest biomass residues derived from *Pinus pinaster*, *Pinus pinea*, and the invasive species *Acacia dealbata*, with a focus on their potential application as bioadsorbents. A comprehensive physicochemical characterization was conducted for different biomass fractions (leaves, needles, and branches of varying diameters). Leaves and needles contained higher amounts of extractives (from 7.7% in acacia leaves to 18.8% in maritime pine needles) and ash (3.4 and 4.2% in acacia leaves and stone pine needles, respectively), whereas branches contained more holocellulose (from 59.6% in *P. pinea* small branches to 79.2% in *P. pinaster* large branches). ATR-FTIR and pHpzc analyses indicated compositional and surface charge differences, with higher pHpzc values in *A. dealbata* relative to Pinus. TG analysis showed that acacia large branches degraded at a lower temperature (320 °C) compared to Pinus species (440–450 °C). Overall, the findings highlight the suitability of these underutilized forest byproducts as bioadsorbents, contributing to the advancement of circular economy practices.

## 1. Introduction

Growing global concerns regarding environmental sustainability have reinforced strategies based on resource efficiency and the principles of the circular green economy. In this framework, biomass plays a leading role as a renewable and versatile resource, with materials previously regarded as waste now being valued for diverse applications, including biosorbents, energy carriers, and chemical sources. Among the various sources of biomass, forest-derived byproducts are particularly relevant, originating both from silvicultural operations and from forest-based industries, especially in countries where these sectors hold significant economic importance. This is the case in Portugal, where forests occupy about one third of the territory and provide raw materials for an economically important forest-based industry [1,2].

Pine species occupy 959 thousand ha, representing 30% of the forest area in Portugal; maritime pine (*P. pinaster*) and stone pine (*P. pinea*) are the most representative and economically significant [3]. *P. pinaster* is mainly exploited to produce timber, directed to saw mills, wood processing mills, particleboard and fiberboard industries with byproducts mainly including small diameter stems from thinning and pruning, and bark. *P. pinea* is valued for pine nuts that have high commercial and nutritional values, and pinecones and shells constitute the major industrial residues [4,5]. Forest residues in Portugal are estimated to amount to approximately 2 million tons annually from silvicultural operations and 5.3 million tons from industrial processing [6]. The identification of alternative valorization pathways, such as their use as bioadsorbents, could represent a sustainable means of generating added value from these secondary resources [7].

In many regions, forests face increasing ecological pressure from invasive species. This is also the case in Portugal, where *Acacia* spp. expanded their distribution area from 2700 ha to 8400 ha between 1995 and 2015 [3]. Initially introduced for stabilization of dunes and soils, as well as for ornamental purposes, they are now widespread and represent a threat to biodiversity and the productivity of native forest species [2,8,9]. Removal initiatives generate large amounts of small dimension stems and branches, and identifying high-value applications for these byproducts could contribute to offsetting removal costs and enhancing the economic feasibility of management strategies [8].

Several forestry and industrial byproducts have been successfully applied as bioadsorbents due to their physicochemical properties, such as high sorption efficiency and the presence of diverse functional groups [10]. Biomass is composed of structural polymers—cellulose, hemicellulose, and lignin—along with pectin, starch, and a variety of organic and inorganic compounds, including phenolic substances and polyphenols. The chemical composition varies depending on the species and plant fraction, resulting in diverse functional groups capable of interacting with metal ions and organic pollutants [11,12]. Accordingly, a detailed preliminary characterization of potential bioadsorbents is critical for their efficient application in adsorption processes, particularly given the heterogeneity of biomass and the variability of its physicochemical properties [12,13].

Previous studies have already demonstrated the successful application of *Pinus* byproducts as biosorbents for heavy metals. Examples include *P. sylvestris* sawdust, *P. brutia* and *P. elliottii* bark, and *P. nigra* and *P. pinea* pinecones, which have been reported as effective in the removal of Pb, Cd, Cr, and Zn from aqueous solutions [14,15,16,17,18]. Relatively to Acacia species, *A. saligna* and *A. nilotica* leaves were studied for the removal of methylene blue and Pb and Cd, respectively [19,20].

This study was undertaken to provide an integrated characterization of residual biomass from commonly found softwood and hardwood species, covering the diversity of biomass types by including distinct fractions of wood and leaves/needles. Thereby, the study addresses the current lack of comprehensive physicochemical descriptions of individual plant components. The primary objective was to elucidate the specific properties of leaves, needles, and branches of two pine species (*P. pinaster* and *P. pinea*) and one acacia (*A. dealbata*), and to evaluate their potential suitability for application as bioadsorbents. The overall goal is to contribute to an economic valorization of forest-based residues that support environmental sustainability and the circular economy.

## 2. Materials and Methods

### 2.1. Materials and Sample Preparation

The analyzed lignocellulosic forestry byproducts were the following:

- branches of maritime pine and stone pine obtained as residues from tree pruning and collected in Viseu, Portugal; the branches had diameters between 1 and 6 cm, and were separated by diameter in two classes: large (4–6 cm) and small (1–3 cm) branches, and processed whole, i.e., wood and bark. The samples were identified as the following: MPLB (maritime pine large branches), MPSB (maritime pine small branches), MPN (maritime pine needles), SPLB (stone pine large branches), SPSB (stone pine small branches), and SPN (stone pine needles).
- Acacia branches obtained from harvested trees, resulting from control and removal operations made in the region of Viseu; the material was processed in the same way as the pine samples, including wood and bark, in two diameter classes: 1–3 and 4–6 cm. The samples were identified as the following: ALB (acacia large branches), ASB (acacia small branches) and AL (acacia leaves).

The materials were dried before milling (Retsch SMI mill, Retsch-Allee, Haan, Germany) and sieving (Retsch AS200 Retsch-Allee, Haan, Germany). Chemical analyses used the 40–60 mesh fraction. Analyses of surface and morphology used the powder fraction (<80 mesh).

### 2.2. Chemical Composition

The chemical composition of the samples included the determination of extractives, holocellulose, α-cellulose, lignin, and ash contents. All analyses were performed in triplicate. The results are reported as the mean percentage, relative to the initial dry mass of the sample.

Determination of the extractives followed the Tappi T 204 standard [21], using successive extractions with dichloromethane, ethanol, and water, and quantification of the dry mass remaining after solvent evaporation and drying.

Holocellulose content was determined by the acid-chlorite method proposed by Browning (1967) [22], while α-cellulose was quantified according to the Tappi T 203 method [23]. Both components were quantified as the residue’s dry mass. The hemicellulose content was estimated as being the difference between the holocellulose and α-cellulose contents.

Lignin content was determined following the Tappi T 222 standard [24]. Insoluble lignin (Klason lignin) was quantified as the dry mass of the solid residue obtained after filtration and drying. Soluble lignin was quantified by measuring the absorbance of the filtrate at 205 nm, using a Perkin Elmer Lambda 25 UV-Visible spectrophotometer (Perkin Elmer, Peterborough, UK) and a sulfuric acid solution (1.2%), as standard. The determination of soluble lignin was carried out with an absorption coefficient of 110 L.g^−1^cm^−1^ in the calculations.

Both lignin and holocellulose analyses were performed on extractive-free samples.

### 2.3. Ash Content and Composition

Ash content was determined by combustion in a muffle furnace at 525 °C until white ash was obtained. Results were expressed as the percentage of ash dry mass, relative to the initial dry mass of the sample.

For cation quantification, the ashes were digested with 65% nitric acid. Samples were heated in crucibles on a heating plate, with acid added gradually until complete digestion was achieved. The samples were filtered with filter paper and diluted to a final volume of 50 mL with distilled water. The concentrations of calcium (Ca), magnesium (Mg), potassium (K), sodium (Na), and zinc (Zn) were determined using flame atomic absorption spectrophotometry (AAS, Washington, DC, USA; Perkin Elmer AAnalyst 300).

### 2.4. Point of Zero Charge

Approximately 0.10 ± 0.05 g of each sample was added to 25 mL of distilled water with adjusted pH values (sodium hydroxide and hydrochloric acid 0.1 M) from 2 to 12 in Erlenmeyer flasks. The pH was monitored with a HACH HQ 30d pH meter (Hach Lange, Düsseldorf, Germany). Samples were agitated at room temperature for 24 h, after which the final pH was recorded. The point of zero charge (pHpcz) was determined within the range where a buffering effect was observed: that is, where the pH remained stable regardless of the initial adjusted pH value.

### 2.5. TGA, XRD, SEM-EDS and ATR-FTIR Analysis

Thermogravimetric analysis (TGA) was carried out using a Mettler V3 thermobalance coupled with a Mettler TC10A processor (Mettler-Toledo AG, Greifensee, Switzerland), employed to determine weight loss following a rise in temperature. The mass loss was monitored throughout the heating process at a rate of 5 °C min^−1^, up to 800 °C. The analysis was made in duplicate.

Powder X-ray diffraction (XRD) data were collected at an ambient temperature on an Empyrean PANalytical diffractometer, with a working wavelength of λ1 = 1.540598 Å and λ2 = 1.544426 Å (Cu Kα1,2 X-radiation), equipped with a PIXcel 1D detector and a flat-plate sample holder in a Bragg–Brentano para-focusing optics configuration (45 kV, 40 mA). Intensity data were collected by the step-counting method (step 0.01°) in continuous mode, in the 3.5° ≤ 2θ° ≤ 50° range. Replicates were performed when the initial analysis yielded unsatisfactory results. The crystallinity index (CI) was calculated according to the method of Segal et al. (1959) [25]:

$$CI(\%)=\frac{I_{002}-I_{am}}{I_{002}}\times 100$$

where I_002_ corresponds to the diffraction intensity near 2θ = 22°, representing the crystalline fraction, and I_am_ corresponds to the diffraction intensity near 2θ = 18°, associated with the amorphous fraction in cellulosic fibers.

Scanning electron microscopy–energy dispersive X-ray spectroscopy (SEM-EDS) was carried out on a high-resolution Hitachi SU-70 (Monocomp, Tokyo, Japan), working at 15 kV. Samples were prepared by deposition on aluminum sample holders, followed by carbon coating using an Emitech K950X carbon evaporator (Emitech Group, Montigny-le-Bretonneux, France). Images with different degrees of magnification were collected. During the EDS analysis of the acacia samples, certain interferences attributable to the preparation process were identified: specifically, the presence of aluminum and tungsten. These elements are likely associated with the sample holder, or with residual contamination from previously analyzed specimens.

ATR-FTIR spectra were obtained in a Perkin Elmer Spectrum Two spectrometer (Perkin Elmer, Peterborough, UK) with 10 scans/min with a resolution of 4 cm^−1^ over the 4000 to 500 cm^−1^ range for the initial materials. The samples were dried in an oven at 100 °C before testing. The spectrum was obtained by placing the sample powder over the crystal and pressing it against the crystal. The average of three spectra was used.

## 3. Results

### 3.1. Chemical Composition, Ash, and Cationic Content

The results obtained for the chemical composition of the samples are presented in Table 1, and those for ash content and composition are presented in Table 2.

Chemical differences were found regarding both species and biomass components. Content in extractives showed significative differences among samples, e.g., large branches contained 4.4%, 12.6% and 5.5%, respectively, for acacia (ALB), stone pine (SPLB), and maritime pine (MPLB). The lignin content was very similar between samples ranging from 31.2% (SPSB) to 39.2% (ASB). The holocellulose content was similar for acacia and maritime pine samples, above the content found for stone pine samples. The α-cellulose content was also notably different, varying from 35.8% (ALB) to 60.5% (MPLB).

The leaves and needles showed the highest extractives content in each species, with the highest value (18.8%) found for maritime pine needles (MPN).

When considering the summative chemical analysis of biomass, a remark should be made, especially when dealing with non-wood lignocellulosic materials with high extractive content, such as bark and leaves. The observed overestimation could be attributed to an incomplete initial extraction of the extractives contained within the sample. Lignin content may be overestimated due to the presence of highly condensed extractives that are not easily solubilized, and therefore, the Klason acid residue may include them. Lignin and extractives may also interfere with the quantification of cellulose and hemicellulose by their incomplete removal, thereby also leading to cellulose and hemicellulose overestimations.

The ash content (Table 2) was different between samples, with the highest ash content found in the leaves/needles, with 3.4% for acacia leaves (AL), 4.2% for stone pine needles (SPN), and 3.5% for maritime pine needles (MPN). The ash content in large branches was lower than in small branches, e.g., for maritime pine, 0.8% and 1.6% (MPBB), and for stone pine, 1.3% and 2.1%.

Regarding mineral composition, for acacia large branches (ALB) the main element was potassium (K) with 1.77 mg g^−1^, while maritime and stone pine large branches (MPLB and SPLB) presented a higher content of calcium (Ca), 1.56 and 3.23 mg g^−1^, respectively. Calcium was also the main element found in the small branches of maritime and stone pines, with values of 1.72 and 5.93 mg g^−1^, respectively. Likewise, leaves and needles presented higher values of calcium. However, in small branches of acacia, the main element found was potassium, with 2.72 mg g^−1^.

### 3.2. Point of Zero Charge

The point of zero charge (pHpzc) was determined as the final pH that was identified in more than one sample. The results are presented in Table 3.

The point of zero charge analyses indicated differences between samples, with higher pHpzc values in *A. dealbata*, relative to Pinus species.

### 3.3. Thermogravimetric Analysis (TGA)

**Figure 1:**
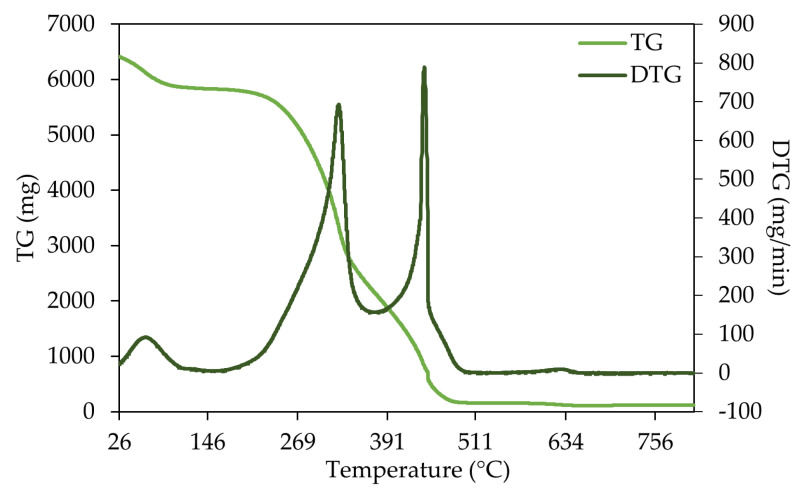
TG and DTG curve of stone pine large branches (SPLB).

**Figure 2:**
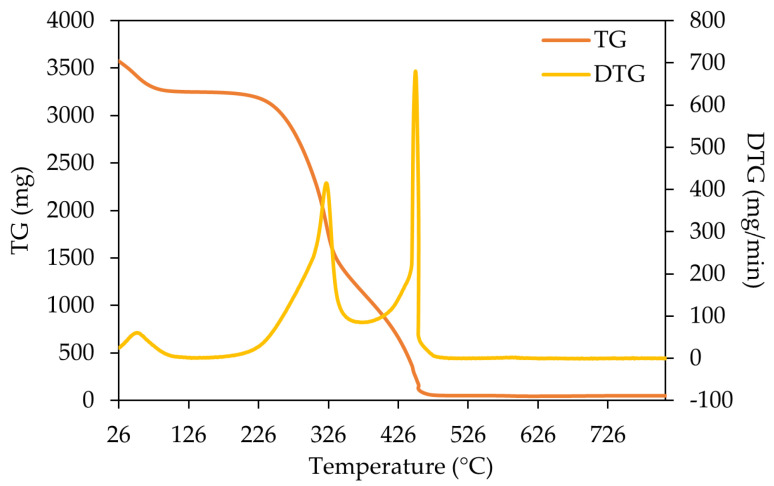
TG and DTG curve of maritime pine large branches (MPLB).

**Figure 3:**
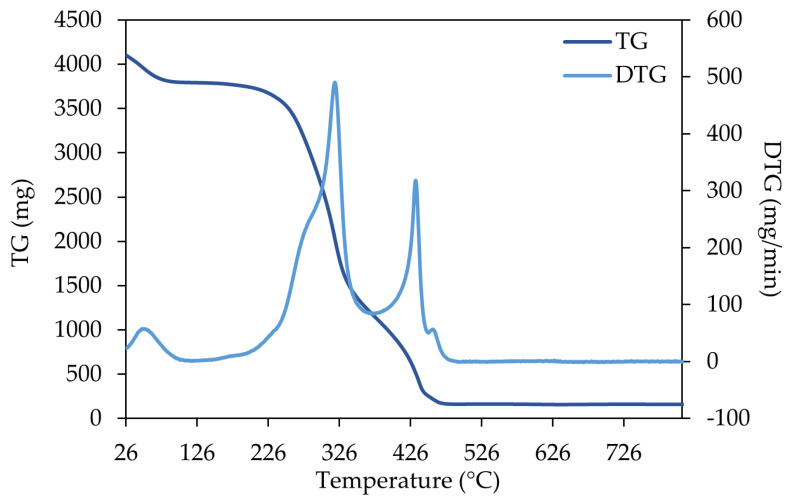
TG and DTG curve of acacia large branches (ALB).

The results of the thermal analysis are presented in Figure 1, Figure 2 and Figure 3 for three representative samples (SPLB, MPLB, and ALB). Their thermogravimetric (TG) and derivative thermogravimetric (DTG) curves highlight differences in thermal decomposition behavior and biomass composition. The thermogravimetric analyses of the remaining samples are presented in the Supplementary Materials (Figures S2–S7).

The thermogram for large branches of stone pine (Figure 1) shows three major mass-loss events: the first stage (~30–130 °C) corresponds to moisture evaporation, the second stage (~200–380 °C) reflects mostly the active devolatilization of hemicellulose and cellulose, and the third region (>400 °C) represents lignin decomposition and slow carbonization. The DTG peaks indicate that a high degradation rate occurred around 440 °C. In the TG curve, it was possible to observe that the devolatilization process begins around 230 °C, becoming more pronounced until ~450 °C.

Comparable thermal behavior was observed for stone pine small branches and needles (SPSB, Figure S2; SPN, Figure S3). Nevertheless, an additional mass-loss event was detected at approximately 600–640 °C, suggesting that dihydroxylation in these samples may not have been fully completed. Furthermore, the second and third mass-loss stages exhibited a more gradual and continuous transition, with the maximum degradation occurring at approximately 325 °C in both cases.

The thermogram analysis of maritime pine large branches (Figure 2) also demonstrated three principal stages of mass loss, comparable to those observed for maritime pine small branches (MPSB) and maritime pine needles (MPN), represented in Figures S6 and S7, respectively. However, the devolatilization of hemicellulose and cellulose, along with the minor lignin degradation characterized by the second peak, was relatively less pronounced in MPLB compared to MPSB and MPN. The degradation of the sample components initiated at ~240 °C, becoming more remarkable until ~460 °C. The DTG curve indicated the maximum degradation was around 450 °C for MPLB and MPSB, whereas for MPN it occurred at ~315 °C.

The thermogram for acacia large branches (Figure 3) shows a more pronounced degradation of polymers between ~220 and 460 °C. The DTG curve shows three accentuated mass-loss events, but a fourth small peak was observed around 460 °C. The first stage was around ~30–100 °C (moisture evaporation); the second between ~200–350 °C (devolatilization and degradation of cellulose, hemicellulose, and lignin), where the maximum degradation rate occurred at 320 °C; and the third, around 400–450 °C (total lignin decomposition).

Similarly, acacia small branches (ASB, Figure S4) exhibited four distinct mass-loss events, displaying a degradation profile comparable to that of ALB. However, the third peak, associated with lignin decomposition, was notably less pronounced in ASB than in ALB. In contrast, acacia leaves (AL, Figure S5) showed five mass-loss events, including three minor peaks between ~430–520 °C, corresponding to the completion of lignin decomposition and dihydroxylation. The TG curve of the leaves indicated a slower degradation profile (~210–520 °C) compared to that of acacia branches.

### 3.4. Powder X-Ray Diffraction (XRD)

**Figure 4:**
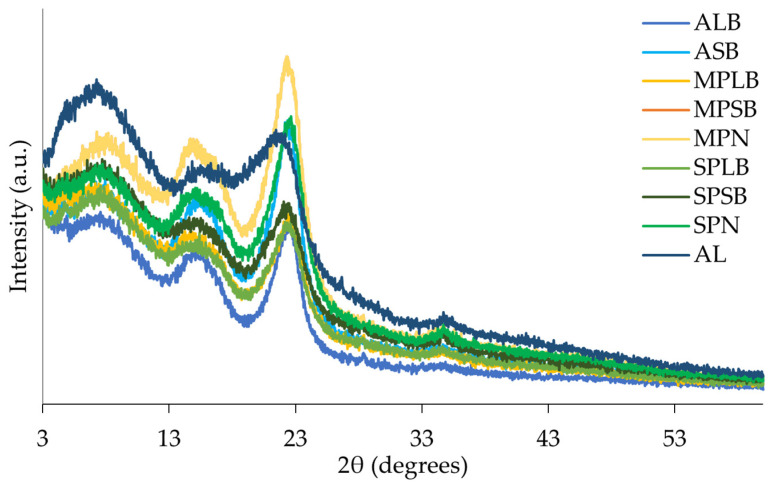
Diffractogram of acacia large and small branches and leaves (ALB, ASB, and AL, respectively), stone pine large and small branches and needles (SPLB, SPSB, and SPN, respectively), and maritime pine large and small branches and needles (MPLB, MPSB, and MPN, respectively).

The diffractograms obtained by the XRD method are presented in Figure 4, for all samples. The calculated crystallinity indices (%) are presented in Table 4.

A characteristic peak around 22–24° is observed in all samples. This peak is related to the cellulose crystalline fraction, which occurs within 22° < 2θ < 23° for cellulose type I and 18° < 2θ < 22° for cellulose type II [26]. Typical powder XRD patterns of plants, including wood, exhibit diffraction peaks centered at approximately 14.9°, 16.7°, 22.9°, and 34.5° [27,28]. The crystalline cellulose is often found between 30 and 35° [27], being identified with less intensity among the samples in this study. The amorphous fraction is observed at 18° < 2θ < 19° for cellulose type I and 13° < 2θ < 15° for cellulose type II [26], and this characteristic peak was also observed in all samples.

The crystallinity index [25] showed differences between samples (Table 4). Acacia samples exhibited greater crystallinity in the branches (36.5% and 33.5% in ALB and ASB, respectively), whereas pine samples (maritime and stone pine) displayed a higher crystallinity index in the needles (34.6% and 31.0% in MPN and SPN, respectively).

### 3.5. Scanning Electron Microscopy–Energy Dispersive X-Ray Spectroscopy (SEM-EDS) Analysis

**Figure 5:**
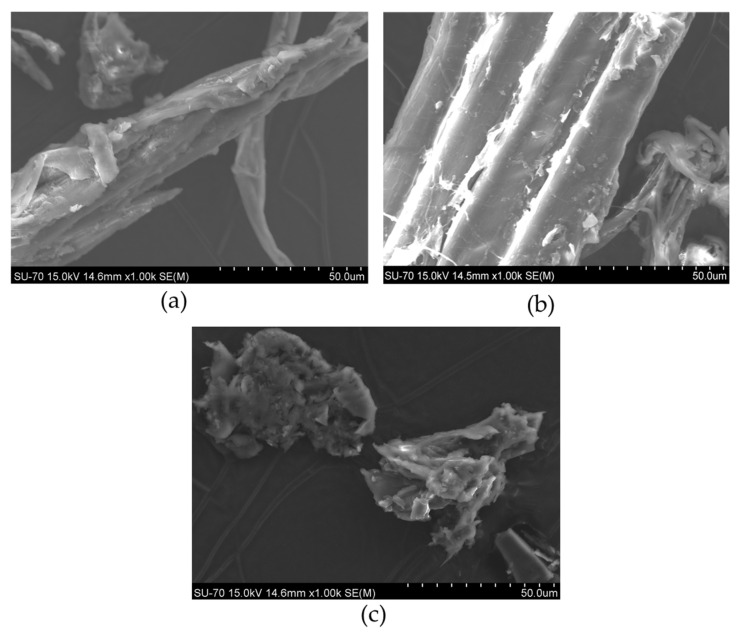
SEM images of acacia large branches (**a**), maritime pine large branches (**b**), and stone pine large branches (**c**). All imagens have a magnification of 1000×.

The micrographs obtained by SEM analysis for acacia, maritime, and stone pine large branches (ALB, MPLB, and SPLB, respectively) are shown in Figure 5. The micrographs recorded for the other materials are shown in the Supplementary Materials (Figure S1). 

Scanning electron microscopy (SEM) revealed cellular fragments and cell wall fractions, with all samples exhibiting irregular and heterogeneous surfaces, often displaying microcracks and porosity. These morphological features are typical of lignocellulosic materials and may result from the intrinsic fibrous structure, drying processes, or surface degradation during sample preparation. Such characteristics can influence the material’s surface reactivity and mechanical stability.

**Figure 6:**
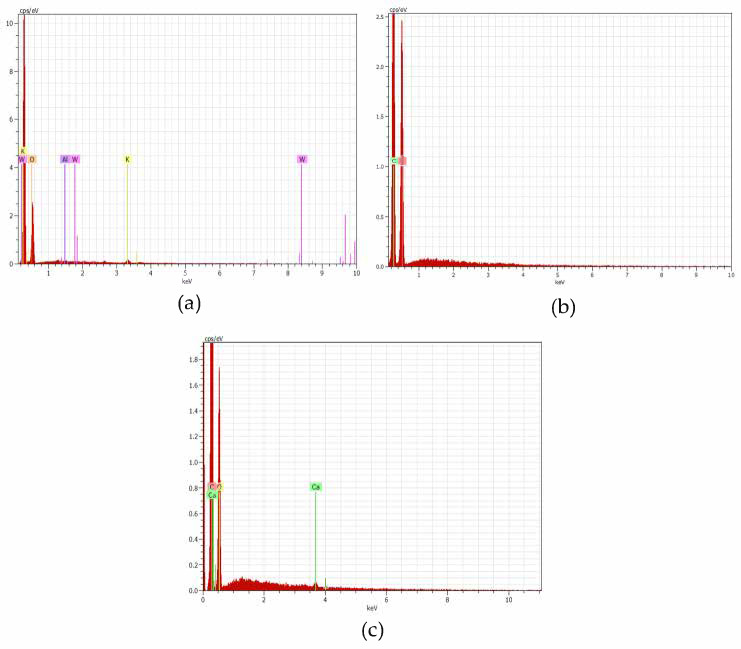
EDS analysis of (**a**) acacia larger branches (ALB), (**b**) maritime pine larger branches (MPLB), and (**c**) stone pine larger branches (SPLB).

The elements identified by the energy dispersive spectroscopy (EDS) analysis are shown in Figure 6, for acacia, maritime pine, and stone pine, respectively.

Energy-dispersive X-ray spectroscopy (EDS) confirmed the organic nature of the samples, with carbon and oxygen being the predominant elements across all species. However, variations in elemental composition were observed, depending on the botanical origin. In acacia (Figure 6a), the main elements detected were oxygen (85.7 wt%) and potassium (10.2 wt%), with minor amounts of tungsten (2.7 wt%) and aluminum (1.3 wt%). The high oxygen content reflects the abundance of hydroxyl and carbonyl groups that are typical of cellulose and hemicellulose. Potassium is likely derived from naturally occurring plant salts. The presence of tungsten and aluminum, which are uncommon in plant biomass, is likely due to external contamination from the sample holder or preparation environment. The maritime pine sample (Figure 6b) showed only carbon and oxygen, with oxygen having the most intense peak. This elemental profile indicates a clean lignocellulosic composition, with no detectable inorganic or metallic impurities. For stone pine (Figure 6c), the EDS analysis revealed carbon (68.4 wt%) and oxygen (31.3 wt%) as the major constituents, along with trace amounts of calcium (0.3 wt%). Calcium may originate from absorbed mineral content or residual environmental particles.

Overall, EDS analysis confirmed the dominance of C and O, as expected in lignocellulosic structures.

### 3.6. Transform Infrared Spectroscopy (ATR-FTIR) Characterization

**Figure 7:**
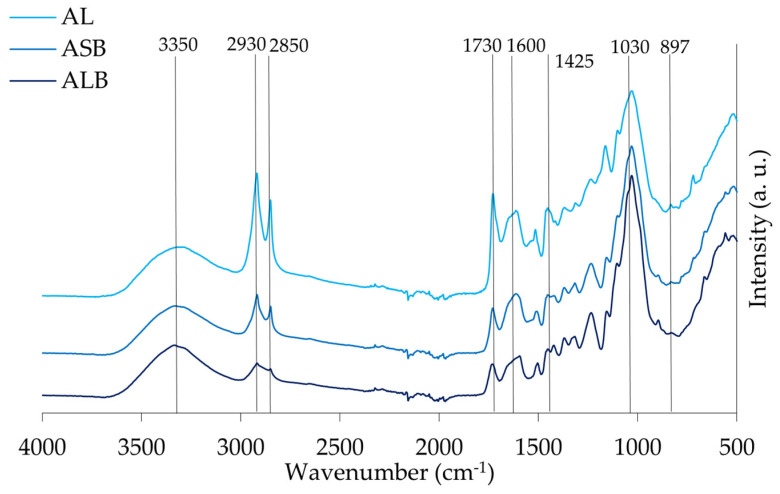
ATR-FTIR spectra of acacia leaves (AL), and acacia large and small branches (ALB and ASB), respectively.

**Figure 8:**
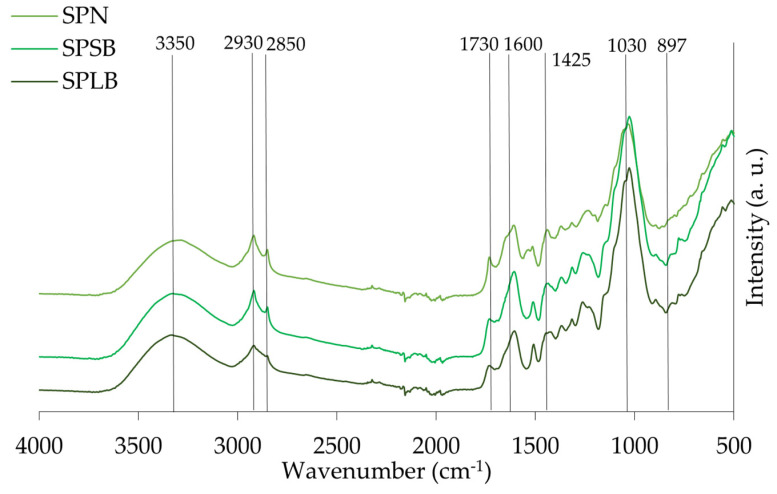
ATR-FTIR spectra of stone pine needles (SPN), and stone pine large and small branches (SPLB and SPSB), respectively.

**Figure 9:**
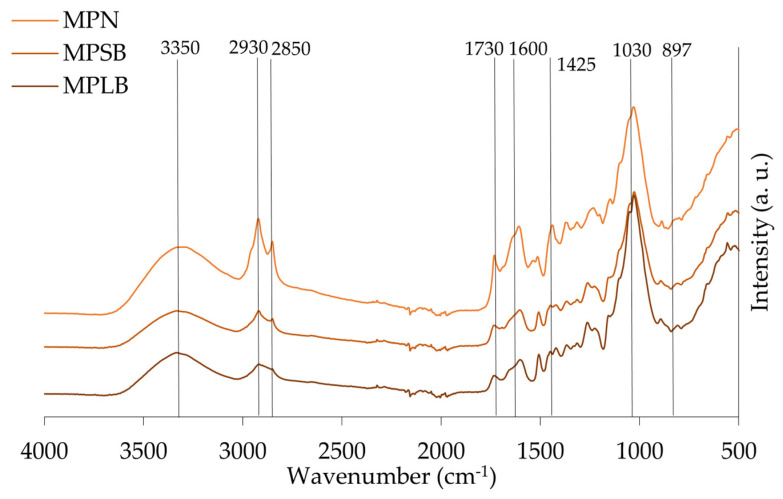
ATR-FTIR spectra of maritime pine needles (MPN), and maritime pine large and small branches (MPLB and MPSB), respectively.

The spectra of the different materials, leaves/needles, small and large branches of stone pine, maritime pine, and acacia are presented in Figure 7, Figure 8 and Figure 9, respectively. Identification of the peaks and their associated functional groups are given in Table 5.

FTIR spectroscopy demonstrated distinct chemical profiles among *A. dealbata*, *P. pinea* (stone pine), and *P. pinaster* (maritime pine) fractions. Acacia leaves exhibited enhanced aliphatic signatures with strong –CH_2_ stretching (2850–2930 cm^−1^) and ester/carboxylic C=O absorption (~1730 cm^−1^), indicative of waxes, fatty acids, and pectins. The fingerprint region (~1030 cm^−1^) confirmed cellulose/hemicellulose predominance, while β-glycosidic linkages (~897 cm^−1^) suggested amorphous cellulose.

Stone pine samples showed relatively uniform compositions, with slightly higher C–H stretching (2930 and 2850 cm^−1^) in needles, reflecting modestly elevated lipophilic compounds. Subtle absorptions at 1640–1550 cm^−1^, along with strong C–O (~1030 cm^−1^) and guaiacyl (~1260 cm^−1^) bands, particularly in branches, indicated higher structural carbohydrate and lignin content.

In maritime pine, hydroxyl levels were consistent across tissues, but –CH_2_ stretching was significantly more intense in needles. Unexpectedly, aromatic C=C stretching (1600, 1505, 1450 cm^−1^) was also stronger in needles, likely reflecting non-structural phenolics, aligning with their elevated extractives (18.8%). Conversely, carbohydrate- and lignin-associated absorptions (1030, 1260 cm^−1^) were more pronounced in branches.

## 4. Discussion

### 4.1. Chemical Composition

The chemical characterization of the *Acacia dealbata*, *Pinus pinea*, and *Pinus pinaster* samples underscored the necessity of species- and tissue-specific analyses, as substantial compositional differences were observed. Leaves and needles were chemically distinct from branches, exhibiting higher extractive (7.7% in acacia leaves; 14.1% in stone pine needles; and 18.8% in maritime pine needles) and ash contents, with calcium as the predominant mineral.

For *A. dealbata*, comparisons with previous studies revealed consistent trends, although some divergences were noted. The literature shows a wood composition of 42.4–50.9% of cellulose, 19.3–25.9% of total lignin, 3.1–8.3% total extractives, and 0.5–1.1% ash content [30,31]. For the leaves and stalks, values of 43.1% cellulose, 25.9% lignin, 8.3% extractives, and 0.5–1.1% ash contents were found [31]. The extractive content found in this work (7.7%) is similar, but the total lignin (53.9%) and ash contents were much higher (3.4%).

Previously, the chemical analysis of *P. pinaster* needles showed an extractive content of 6.6% [29], below the values found in this work for maritime pine needles (18.8%) and stone pine needles (14.1%), and also a lower ash content (1.2%). However, an ash content of 4.4% was reported, as well as a holocellulose content of 51.6%—very similar to the values found in this work—and a lignin content of 43.2%, which were higher than the values found in needles in this study [32].

A study on the chemical components of *P. pinaster* wood from Turkey reported a holocellulose content of 72.1 to 82.2%, α-cellulose between 47.7 and 53.2%, total lignin of 26.4 to 28.3%, and ash 0.31 to 0.38% [33]. The holocellulose and α-cellulose contents were similar to the results found in this work for small and large branches of *P. pinaster*, although the total lignin and ash content were higher. For *P. pinea* wood, the literature exhibits an ash content ranging from 0.2 to 0.3%, total lignin between 27.7 and 30.0%, holocellulose around 61.1–64.3%, α-cellulose of 43.1–47.5%, and hemicellulose between 16.8–18.5% [34]. In this work, very similar values were found for holocellulose and α-cellulose, but lignin and hemicellulose contents were higher, as well as the ash content. Additionally, smaller branches (MPSB, SPSB), which contain proportionally more bark, demonstrated higher extractive and ash contents, reinforcing the influence of anatomical composition on chemical variability [35,36].

The chemical structural composition of biomass (cellulose, hemicellulose, and lignin) plays a crucial role in the sorption process, as all these biopolymers contain functional groups that are relevant for contaminant binding. Cellulose provides hydroxyl (–OH) groups that contribute to hydrogen bonding, whereas hemicellulose offer a higher density of active sites, due to their greater accessibility, and the presence of additional functional groups such as ester and carboxyl moieties. Lignin contains methoxyl, phenolic, and carboxyl groups, which can interact with and effectively bind contaminants.

Ash content exerts a dual effect on sorption. On one hand, the deposition of inorganic minerals within material pores may obstruct porosity and reduce the accessible surface area, thereby limiting sorption capacity. On the other, ash constituents with intrinsic porosity or a high surface area may enhance sorption performance. The influence of calcium and potassium is particularly relevant, due to their involvement in ion-exchange mechanisms. These cations may compete with target species for binding sites, altering sorption dynamics and potentially decreasing uptake efficiency; elevated concentrations in aqueous media can further hinder the sorption of competing cations [37].

Although some studies on macroalgae, microalgae, and cyanobacteria reported no direct correlation between calcium and potassium concentrations and sorption capacity [37], other evidence suggests that calcium-containing phases—such as calcium oxalate monohydrate in *Eucalyptus globulus* biochar—can reduce sorption efficiency (e.g., of bisphenol A) through pore blockage and decreased surface accessibility [38]. Experimental results also indicate that the removal of ash may reopen obstructed pores; however, this does not necessarily improve sorption kinetics, as the structural characteristics of porous ash residues may impose additional limitations.

### 4.2. Point of Zero Charge (pHpzc)

The point of zero charge (pHpzc) reflects the balance between positive and negative surface charges and depends on both the material’s functional groups and the solution pH [39].

In this study, pHpzc values ranged from 3.95 (SPN, MPSB) to 5.29 (ASB), consistent with reported values for maple sawdust (4.72), peat moss (3.42) [39], rubber wood shavings (4.35) [40], and *Cuminum cyminum* waste (5.70) [41]. Additional evidence from soil systems demonstrated that the net surface charge decreases with a rising pH, while pHpzc varies across soil types [42]. Our results aligned with this trend, with variations among lignocellulosic byproducts indicating the influence of chemical composition on surface charge behavior.

The pH plays a pivotal role when considering a possible application in adsorption, since it significantly influences the ionization degree of the chemical species, the surface charge of the adsorbent, and the molecular configuration of the adsorbates, modulating the nature of the interactions between adsorbent and adsorbate by affecting the ionization states of the involved species [41].

Some studies have shown that inorganic pollutants, such as metal ions, are mostly removed from aqueous solutions in acid or neutral pH [43,44]. When pH < pHzpc, the biosorbent surface develops a positive charge, repelling positively charged ions, while the opposite effect (negative charge) is observed when pH > pHzpc, attracting positively charged ions. Thus, adsorption is favored when pHzpc is below the ideal pH for removing the desired contaminant.

### 4.3. TGA, XRD, SEM-EDS, and ATR-FTIR

#### 4.3.1. Thermogravimetric Analysis (TGA)

Thermogravimetric analysis (TGA) provides a real-time assessment of biomass mass loss during heating, generating TG and DTG curves that represent weight reduction and its rate, respectively. The thermal behavior of lignocellulosic biomass is strongly influenced by chemical composition, particularly the relative proportions of cellulose, hemicellulose, and lignin [45,46].

Across all samples, three main degradation stages were identified: (i) moisture evaporation; (ii) active devolatilization of hemicellulose and cellulose, accompanied by partial lignin degradation; and (iii) complete lignin decomposition. These results align with reported profiles for *P. sylvestris* and *P. pinaster*, which exhibit three main phases—water evaporation (20–150 °C), pyrolytic degradation (188–426 °C), and carbonization (>500–1017 °C) [33]—and also for *Populus L.*, which follows similar steps [47].

In this study, SPLB and MPLB samples (*P. pinea* and *P. pinaster* branches) exhibited degradation patterns typical of softwoods, with DTG peaks occurring between 310 and 500 °C, reflecting cellulose and lignin decomposition. Comparable maximum decomposition rates (~310 °C) have been reported for pine fibers, while other natural fibers showed lower maxima (~290 °C) [48].

Conversely, ALB (*Acacia dealbata* branches) degraded at lower temperatures, consistent with hardwoods [49] characterized by lower lignin content and higher proportion of thermolabile hemicellulose, resulting in reduced thermal stability. Residual mass analysis further supported these differences: SPSB retained more carbon, while ALB showed lower residues, underscoring the greater carbon retention potential of pine-derived biomass, which is particularly relevant for biochar production.

Lignin content plays a decisive role when considering biomass utilization for biochar production. Biomass with higher lignin fractions, like maritime pine branches, generally produces more stable biochar, as lignin is more resistant to thermal degradation and retains greater amounts of carbon, resulting in a higher fixed carbon content and enhanced long-term stability [50]. In contrast, cellulose and hemicellulose yield more volatile products during pyrolysis, leading to biochar with a lower fixed carbon content. This is the case of acacia branches with a hemicellulose content of 43.2% (ALB) and 36.0% (ASB). Fixed carbon is a critical quality parameter, representing the thermally stable fraction that remains after pyrolysis [50]. The decomposition temperature of cellulose, hemicellulose, and lignin is different, and the corresponding mass loss during each stage reflects their relative abundance, thereby enabling compositional comparisons between materials.

Mineral constituents, including potassium, calcium, and manganese, also influence biochar properties. While these elements may enhance cation exchange capacity, they can simultaneously destabilize biochar structure. Moreover, their presence generally increases the final biochar pH, rendering it more alkaline, which can further affect its sorption behavior. Large amounts of potassium were observed in acacia branches, while pine branches showed higher levels of calcium.

#### 4.3.2. Powder X-Ray Diffraction (XRD)

X-ray diffraction (XRD) is a widely employed technique for assessing material crystallinity or crystallinity fractions, parameters that are strongly influenced by the chemical composition of the samples [51,52], and domains are defined by a regular three-dimensional molecular arrangement, reflecting the long-range structural order [53]. Given that cellulose constitutes more than 50% of the dry mass of wood, wood diffractograms are largely representative of cellulose diffraction patterns [52,54]. Hemicellulose and lignin are predominantly amorphous, whereas cellulose contains both amorphous and crystalline regions [55], which helps to explain the mainly amorphous nature of the studied samples.

In this study, three main broad diffraction peaks were consistently identified across all samples: the first at approximately 10°, the second between 15 and 18°, and the third in the range of 20–23°. In some samples, a fourth low-intensity peak was observed around 34°. Peaks between 30 and 35° and ~20 and 26° (2θ) are indicative of crystalline cellulose [24,49]. These findings confirm the predominance of amorphous phases with minor crystalline contributions, consistent with previous reports for *P. elliotii* [56], *P. pinaster* bark [11], *Eucalyptus globulus*, and Norway spruce [54]. By contrast, amorphous cellulose in poplar wood was shown to generate broad peaks at 20.5°, 38.9°, and 80.9° (*2θ*) [27].

The X-ray diffraction analysis and crystallinity index of the samples studied correspond to their chemical composition. Acacia branches exhibited higher holocellulose and α-cellulose and lower lignin contents compared to acacia leaves (Table 1), explaining the higher crystallinity values observed in branches relative to leaves (Table 4). In maritime pine, needles presented lower contents of lignin, hemicellulose, and cellulose compared to branches, which may account for their higher crystallinity. A similar pattern was observed in stone pine samples. These values are comparable to those reported for *Picea abies* wood powder (~23–32%) [55], and for maritime pine bark samples, with crystallinity values of ~25.5% for holocellulose and cellulose, and ~41% for α-cellulose [11].

From an adsorption perspective, crystallinity is a critical factor, as it determines solid-phase organization and accessibility to sorption sites. High crystallinity is typically associated with reduced surface area and a lower number of available active sites, while also influencing diffusional pathways of adsorbates, thereby affecting both adsorption kinetics and capacity. In cellulose-based systems, crystallinity directly modulates the accessibility of internal sites to water and metal ions. Lower crystallinity enhances adsorption efficiency [57]. The XRD results of this work confirm the predominantly amorphous character of the studied biomasses, with minor crystalline contributions: a feature that can potentially enhance their sorption performance.

#### 4.3.3. Scanning Electron Microscopy–Energy Dispersive X-Ray Spectroscopy (SEM-EDS)

Scanning electron microscopy (SEM) is a well-diffused method to characterize the surface morphology of lignocellulosic materials [58]. In this work, all the lignocellulosic materials have shown an irregular and heterogeneous surface that resulted from the cellular features and composition of the samples.

The wood anatomy of pine wood, as of most softwoods, is dominated by the presence of tracheid, as identified in the present work (Figure 5). Hardwoods have a more complex anatomical structure, including different cell types, e.g., fibers, vessels, and parenchyma. Depending on trituration and particle size, cellular fragments or clusters are observed. The presence of microcracks and porosity was also observed.

Energy dispersive X-ray spectroscopy (EDS) analysis confirmed that all samples are predominantly composed of carbon and oxygen, consistent with their lignocellulosic nature, while minor elemental variations—such as potassium in acacia and calcium in stone pine—reflect differences in botanical origin.

The morphology of the surface is a critical key in the biosorption process, since a rougher surface is related to an increased surface area [58]. Larger surface areas are related to a greater availability of active sites for adsorption. Likewise, heterogeneous surfaces can favor multilayer sorption, increasing the retention capacity of the material.

#### 4.3.4. Transform Infrared Spectroscopy (ATR-FTIR)

A broad absorption band around 3350 cm^−1^ was observed in all samples. It is attributed to O–H stretching vibrations from hydroxyl groups in cellulose, hemicellulose, and lignin [11], and is commonly reported in lignocellulosic plant materials [59].

A comparative analysis of branches from stone pine (SPSB), maritime pine (MPSB), and acacia (ASB) revealed characteristic lignocellulosic features, including C–H aliphatic chain vibrations (2930 and 2850 cm^−1^), with ASB showing slightly stronger intensities due to its higher hemicellulose and lignin content (Table 2). The peak at 1730 cm^−1^, related to esterified hemicellulose, was most defined in ASB, while C–O stretching (1030 cm^−1^) and β-glycosidic linkages (897 cm^−1^) were most intense in ASB and MPSB, aligning with their respective holocellulose and cellulose content. The α-cellulose content of ASB (37.0%) and SPSB (36.5%) is nearly identical, supporting similar hydrogen bonding environments, while the substantially higher α-cellulose in MPSB (54.7%) does not proportionally increase this peak, suggesting more crystalline or less accessible hydroxyl groups. Differences in aromatic skeletal bands further supported species-specific lignin composition, especially in ASB. Additionally, the small branches presented more intensity in –CH_2_ and C=O peaks compared to larger branches, due to their higher proportion of bark, which includes cork layers that are chemically characterized by the presence of the aliphatic polyester suberin macromolecule.

An analysis of different maritime pine fractions showed a higher presence of extractives and non-structural compounds in the needles (MPN), as indicated by pronounced peaks at 1730 cm^−1^ and 1600 cm^−1^, consistent with the known protein richness of needles [60]. Despite its lower lignin content, MPN exhibited stronger aromatic bands, suggesting contributions from soluble phenolics. In contrast, MPLB showed stronger polysaccharide-related peaks at 1030 cm^−1^ and 897 cm^−1^, consistent with its higher holocellulose content (79.2%). The same trend was observed in stone pine, where large branches (SPLB) exhibited a greater carbohydrate content than small branches (SPSB) and needles (SPN), reinforcing the correlation between tissue type and structural polymer composition. Softwood-specific guaiacyl peaks (1270 cm^−1^) varied among stone and maritime pines.

Acacia samples displayed the most distinct chemical diversity. AL showed stronger aliphatic –CH_2_ bands and ester-related peaks (1730 cm^−1^), suggesting high cuticular wax and pectin content [61]. The band at 897 cm^−1^, which is associated with β-glycosidic linkages typically found in cellulose and hemicellulose, is clearly present in all samples and is particularly prominent in ALB. The strong intensity of this band in ALB indicates a substantial presence of polysaccharide structures. In the fingerprint region, the strong and sharp band at 1030 cm^−1^ is evident in all spectra, with the highest intensity recorded in ALB, followed by ASB. Lignin-specific vibrations (1600 cm^−1^, 1508 cm^−1^) can be observed in all the materials, but with less intensity in ALB, due to the syringyl units in hardwood lignin.

The surface functional groups of lignocellulosic materials play a critical role in adsorption phenomena by governing the affinity and retention of contaminants through both chemical and physical interactions. As discussed in Section 4.1, the principal adsorption-related functionalities, namely hydroxyl and carboxyl groups, were consistently identified across all samples, while others, including aliphatic and carbonyl groups, may further enhance sorption processes by providing supplementary active sites.

## 5. Conclusions

The physicochemical characterization of *Acacia dealbata*, *Pinus pinaster*, and *Pinus pinea* biomass fractions (branches and leaves/needles) revealed distinct compositional and structural differences that were reflected in the thermal degradation profiles and crystallinity indices. Leaves and needles had high extractives and ash contents, whereas branches were enriched in cellulose and hemicellulose, biomass derived from pines demonstrated superior thermal stability, lower crystallinity, and pHpzc values compared to acacia-derived materials.

In general, the investigated lignocellulosic matrices—ALB, ASB, AL, MPLB, MPSB, MPN, SPLB, SPSB, and SPN—were characterized by heterogeneous and irregular surface morphologies, pHpzc values below 5.5, moderate crystallinity indices, and the presence of chemically active functional groups, such as hydroxyl and carboxyl moieties, which are of relevance for adsorption processes. Collectively, these findings highlight that these raw biomasses are potential adsorbent materials in their native state. The production of biochar also emerges as a viable valorization pathway, except for leaves/needles, whose elevated ash content—particularly when enriched in calcium and potassium—may compromise their efficacy in contaminant sequestration from aqueous systems.

## Figures and Tables

**Table 1:** Determination of the chemical composition (% dry matter) of the samples, acacia large and small branches, and leaves (ALB, ASB and AL, respectively), stone pine large and small branches and needles (SPLB, SPSB and SPN, respectively), and maritime pine large and small branches and needles (MPLB, MPSB and MPN, respectively).

	Sample								
Chemical Component (%)	ALB	ASB	AL	SPLB	SPSB	SPN	MPLB	MPSB	MPN
Total extractives	4.4	2.8	7.7	12.6	13.9	14.1	5.5	10.0	18.8
Dichloromethane extractives	0.7	0.7	0.9	2.0	4.8	4.6	2.0	3.5	5.9
Ethanol extractives	2.8	1.2	4.2	9.5	6.9	6.7	2.3	4.2	8.1
Water extractives	0.9	0.9	2.7	1.1	2.2	2.7	1.3	2.3	4.8
Total lignin	34.5	39.2	53.9	31.5	31.2	30.1	38.1	36.6	29.0
Klason lignin	33.9	32.5	49.7	31.2	30.8	28.5	37.1	35.8	28.1
Soluble lignin	0.5	0.5	4.2	0.2	0.3	1.6	1.0	0.9	0.8
Holocellulose	78.9	73.0	57.1	66.8	59.6	57.1	79.2	73.7	64.4
α-cellulose	35.8	37.0	27.7	44.1	36.5	37.8	60.5	54.7	49.8
Hemicellulose	43.2	36.0	29.4	22.6	23.1	19.3	18.7	19.0	14.6

**Table 2:** Ash content and cationic composition of acacia large and small branches and leaves (ALB, ASB, and AL, respectively), stone pine large and small branches and needles (SPLB, SPSB, and SPN, respectively), and maritime pine large and small branches and needles (MPLB, MPSB, and MPN, respectively).

Sample	Ash Content (%)	Cationic Content (mg g^−1^)				
		Ca	Na	Mg	Zn	K
ALB	1.1	0.81	0.23	0.49	0.002	1.77
ASB	2.3	1.42	0.17	1.01	0.01	2.72
AL	3.4	6.02	0.29	1.87	0.003	3.75
SPLB	1.3	3.23	0.07	0.60	0.0003	0.21
SPSB	2.1	5.93	0.26	1.24	0.002	0.70
SPN	4.2	7.11	0.61	1.35	0.002	1.08
MPLB	0.8	1.56	0.18	0.41	0.002	0.37
MPSB	1.6	1.72	0.17	0.63	0.002	0.64
MPN	3.5	0.98	0.14	0.61	0.001	0.32

**Table 3:** Point of zero charge (pHpzc) determined for acacia large and small branches and leaves (ALB, ASB, and AL, respectively), stone pine large and small branches and needles (SPLB, SPSB, and SPN, respectively), and maritime pine large and small branches and needles (MPLB, MPSB, and MPN, respectively).

Sample	pHpzc
SPLB	4.53
SPSB	4.70
SPN	3.95
MPLB	4.84
MPSB	3.95
MPN	4.44
ALB	4.90
ASB	5.29
AL	4.73

**Table 4:** Crystallinity index (%) of acacia (ALB, ASB, and AL), stone pine (SPLB, SPSB, and SPN), and maritime pine (MPLB, MPSB, and MPN) samples, calculated by [25] method.

Sample	IC (%)
ALB	36.3
ASB	33.5
AL	19.6
MPLB	26.2
MPSB	23.1
MPN	34.6
SPLB	27.4
SPSB	25.5
SPN	31.0

**Table 5:** Identification of the peaks. Adapted from [11,29].

Assignment		Peaks Identified in the Samples		
Group	Range (cm^−1^)	ALB	MPLB	SPLB
O-H stretching of hydroxyl group	3336	3331	3331	3337
CH_2_ asymmetric and symmetric stretch from the methyl (CH_3_) and methylene (CH_2_) groups	2916–2936 and 2843–2863	2918 2850	2919 2851	2919 2852
C=O stretch in unconjugated ketones, carbonyls, and ester groups	1738	1731	1733	1735
Identified as C=O stretch and lignin peaks	1603–1608 and 1508–1510	1607 1506	1607 1511	1604 1509
C=C and C-H bond in plane deformation in lignin and hemicellulose	1450–1453	1455	-	1450
Symmetric stretching bands of the carboxyl group associated with CH deformation (methyl and methylene), CH_2_ bending vibrations, and CH_3_ stretching.	1436–1319	-	1435	-
C-OC aromatic ethers, asymmetric stretch	1210–1310	1236	1263	1264
C-O, CC, and C-CO stretch in cellulose, hemicellulose, and lignin	1025–1035	1031	1028	1028
Associated with stretching vibrations of C=O in carboxyl, as well as vibrations related to the aromatic skeleton, usually found in cellulose	738–1614	897	895	895

## Data Availability

The original contributions presented in this study are included in the article. Further inquiries can be directed to the corresponding authors.

## Supplementary Materials

The following supporting information can be downloaded at: https://www.mdpi.com/article/10.3390/ma18204716/s1. Figure S1: SEM micrograph of acacia leaves (a), acacia smaller branches (b), maritime pine needles (c), maritime pine small branches (d), stone pine needles (e), and stone pine bigger branches (f). The AL image has a magnification of 1500×, ASB has a magnification of 200×, MPN has a magnification of 1000×, MPSB and SPN images have a magnification of 500×, and SPBB has a magnification of 1200×; Figure S2: TG and DTG curves of stone pine smaller branches (SPSB); Figure S3: TG and DTG curves of stone pine needles (SPN); Figure S4: TG and DTG curves of acacia smaller branches (ASB); Figure S5: TG and DTG curves of acacia leaves (AL); Figure S6: TG and DTG curves of maritime pine smaller branches (MPSB) and Figure S7: TG and DTG curves of maritime pine needles (MPN).
