# Supplementary material for: Characterization of Lignocellulosic Byproducts from the Portuguese Forest: Valorization and Sustainable Use
Source: Materials (Basel). 2025 Oct 14;18(20):4716. doi: 10.3390/ma18204716 (PMC12566366; doi:10.3390/ma18204716)
Supplement: Supplementary file 1 [file materials-18-04716-s001.zip › materials-3882779-supplementary.pdf]

SEM Images

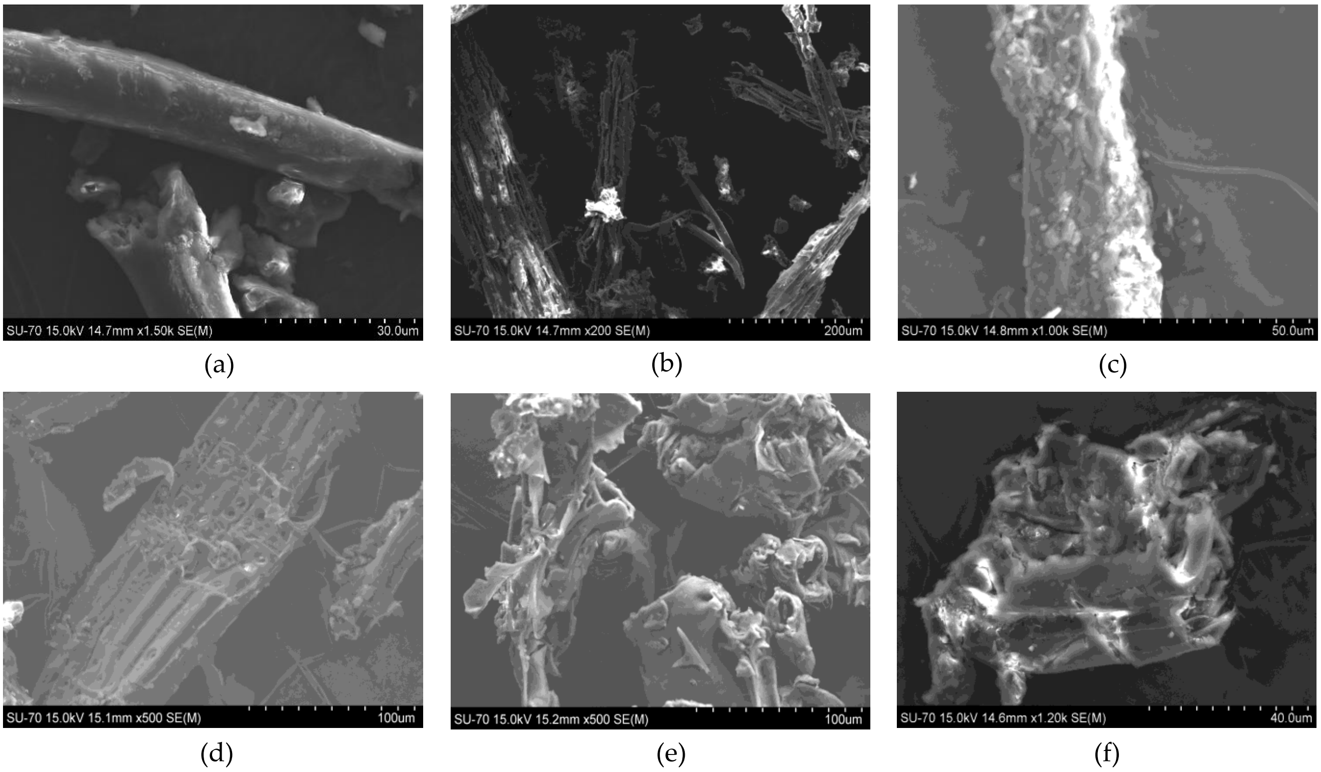

Figure S1.SEM micrograph of acacia leaves (a), acacia smaller branches (b), maritime pine needles (c), maritime pine small branches (d), stone pine needles (e), and stone pine bigger branches (f). The AL image has a magnification of 1500×, ASB has a magnification of 200×, MPN has a magnification of 1000×, MPSB and SPN images have a magnification of 500×, and SPBB has a magnification of 1200×.

Thermogravimetric analysis (TG and DTG curves)

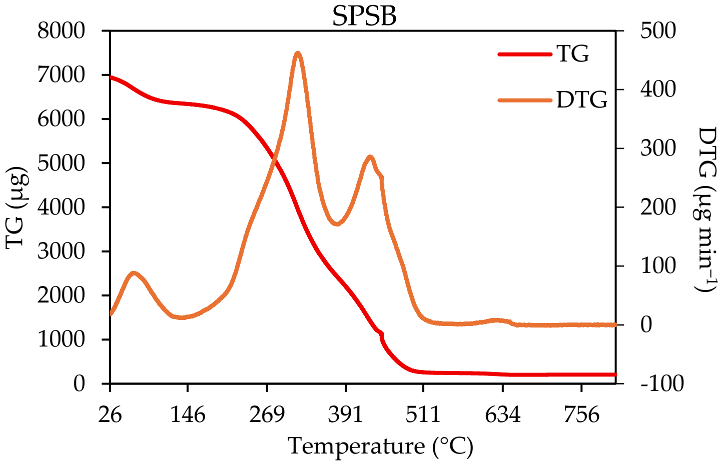

Figure S2. TG and DTG curves of stone pine smaller branches (SPSB).

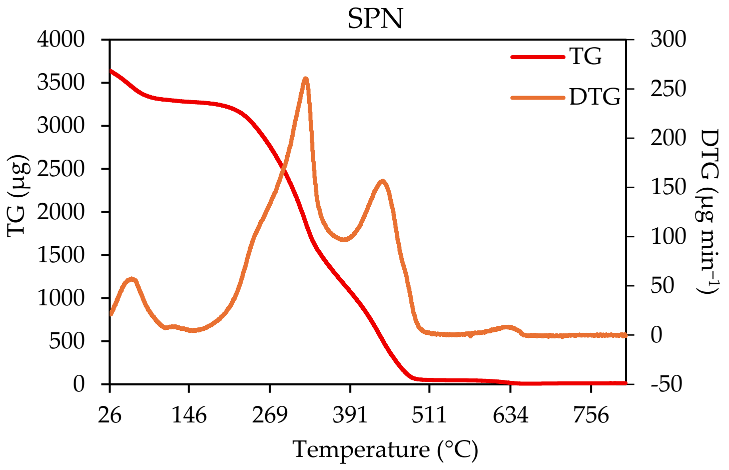

Figure S3. TG and DTG curves of stone pine needles (SPN).

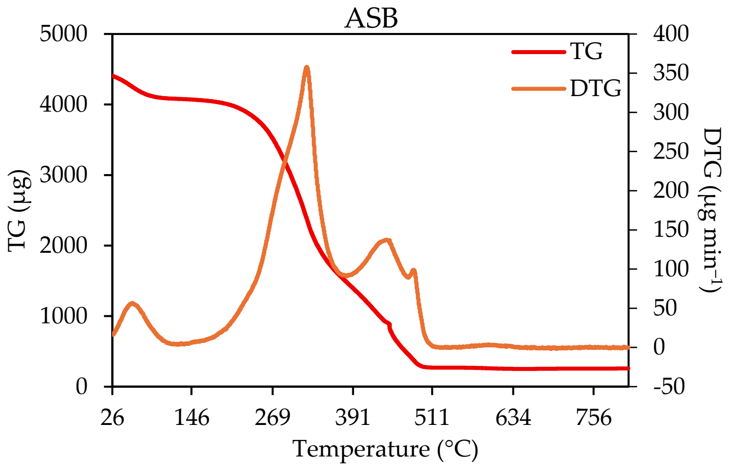

Figure S4. TG and DTG curves of acacia smaller branches (ASB).

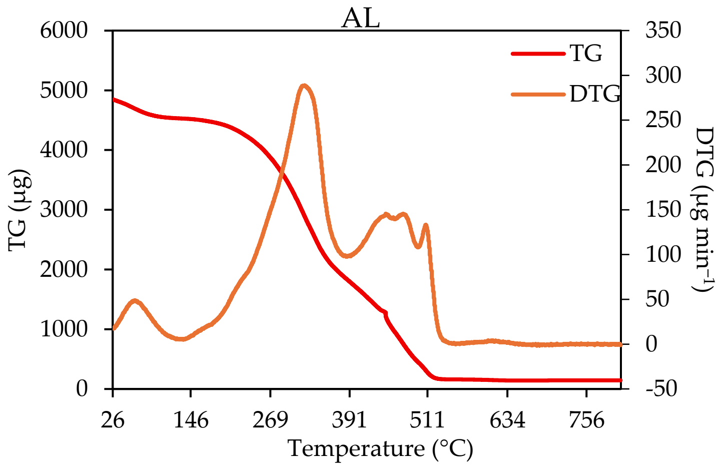

Figure S5.TG and DTG curves of acacia leaves (AL).

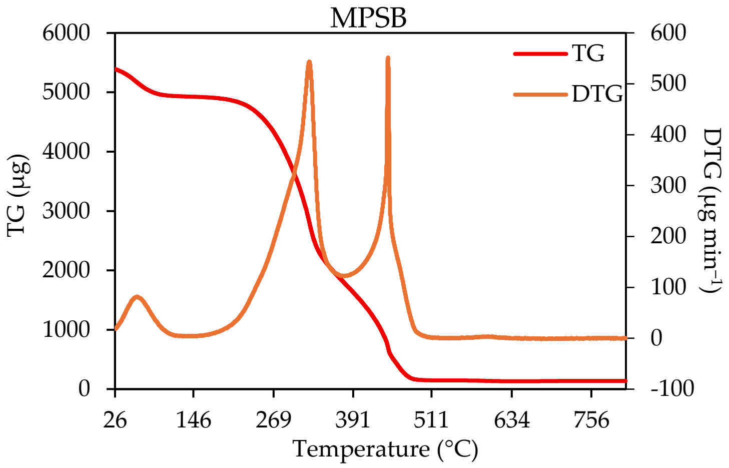

Figure S6. TG and DTG curves of maritime pine smaller branches (MPSB).

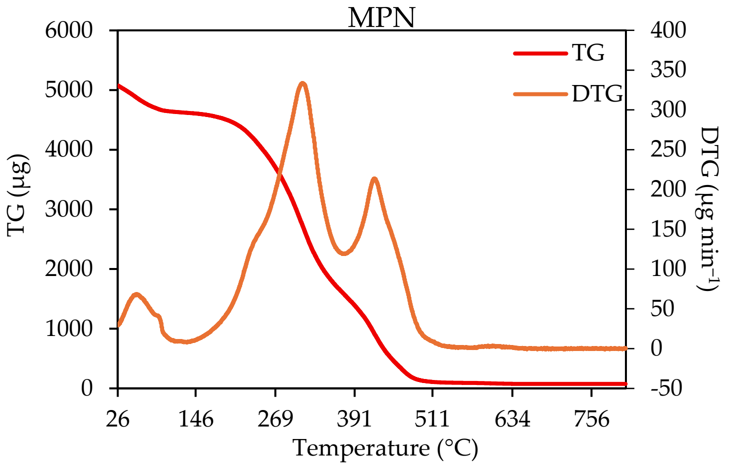

Figure S7. TG and DTG curves of maritime pine needles (MPN).
